# Increasing prevalence of genitourinary schistosomiasis in Europe in the Migrant Era: Neglected no more?

**DOI:** 10.1371/journal.pntd.0005237

**Published:** 2017-03-16

**Authors:** Niccolò Riccardi, Francesca Nosenzo, Francesca Peraldo, Francesca Sarocchi, Lucia Taramasso, Paolo Traverso, Claudio Viscoli, Antonio Di Biagio, Lorenzo E. Derchi, Andrea De Maria

**Affiliations:** 1 Department of Health Sciences (DISSAL), Infectious Disease Section, IRCCS AOU San Martino-IST, Genoa, Italy; 2 Department of Health Sciences (DISSAL), Radiology Section, University of Genoa, Genoa, Italy; 3 Department of Surgical Science (DISC), Luciano Giuliani Department of Urology, University of Genoa, IRCCS AOU San Martino-IST, Genoa, Italy; 4 Department of Surgery (DISC), Pathology Section, University of Genoa, Genoa, Italy; 5 Department of Health Sciences (DISSAL), Radiology Section, University of Genoa, Emergency Radiology, IRCCS AOU San Martino-IST, Genoa, Italy; George Washington University, UNITED STATES

## *Schistosoma haematobium*: An old neglected disease—No more?

Schistosomiasis is a trematode infection that is still spreading through Africa [1,2,3]. It is responsible for urogenital (*S*. *haematobium* [SH]) and intestinal/hepatic (*S*. *mansoni* and other species) complaints and chronic morbidity [4]. Currently, more than 700 million people live in endemic areas [3]. The World Health Organization’s strategic plan for 2012–2020 for schistosomiasis is aimed at controlling morbidity by 2020, eliminating SH infection as a public health problem, and interrupting transmission in select areas by 2025 [5]. These plans are, however, in peril, owing to changes in the epidemiology of SH infection over the last two years. First, campaigns aimed at controlling schistosomiasis through mass drug administration (MDA) have recently been shown to fail at reaching the target population. In 2012, a coverage rate of only 16% of patients needing treatment for schistosomiasis was reported [6]; lack of coverage was reported in 44% of the study population in 2016 [7]. Health education programs and water snail (intermediate host) eradication campaigns, although proven to be effective in reducing the risk of new infections from the 1960s to the 1980s, are no longer actively pursued [5]. Second, the recent rise in the northbound migratory flux from Africa through the Mediterranean basin is steadily contributing to an expansion of the areas where schistosomiasis may be diagnosed. Indeed, within the current migratory flux to Italy and other Mediterranean countries, the most representative nations of origin (The Gambia, Mali, Nigeria, and Sudan) are countries with high endemicity of urogenital schistosomiasis [8,9,10].

## *S*. *haematobium* presentation at the emergency department and increasing referral to urology and radiology—Urine test no more?

This expansion of urogenital schistosomiasis may lead to a lack of recognition of the symptoms of the disease in countries with a high inflow of migrants and where schistosomiasis is only rarely encountered [11]. In nonendemic northern Mediterranean and European countries, which are targets of the migratory flux, the symptoms of urogenital schistosomiasis (e.g., hematuria, urinary frequency, burning micturition, and suprapubic discomfort) may be misinterpreted by the first physician that examines the patient in the emergency department (ED). ED physicians may prescribe a workup that is generally prescribed for European patients, in whom such symptoms primarily suggest complicated cystitis/pyelonephritis or a urinary tract neoplasm. Hence, chronic schistosomiasis occurring in young adult migrants may initially be misdiagnosed or, rather, recognized with delay. Parasitological methods to detect SH eggs in urine and reagent strips to detect microhematuria are very helpful diagnostic tools in endemic areas, but they unfortunately lack the sensitivity to detect mild infections [12]. Moreover, these methods are heavily operator-dependent and involve observer-dependent personal skills. The introduction of recently described highly sensitive and specific diagnostic tests in clinical practice will be needed to improve diagnostic precision in controversial cases [13]. Thus, in developed countries, the combination of the increasing migratory flux from endemic areas and the failing coverage of preventive MDA programs in Africa [7] may result in patients presenting with symptomatic urogenital schistosomiasis to medical departments or surgical wards in which this condition is not immediately recognized. The presentation/referral of a series of consecutive patients who came to the ED of our university teaching hospital in Genoa (northern Italy) confirms this clinical pattern.

We reviewed the clinical history, laboratory findings, and imaging findings in a series of eight male patients (migrants from West Africa) who were consecutively encountered in our practice from July 2015 to January 2016. Patients were first seen by an ED physician and then referred to different wards or services according to their symptoms. The decision based on the clinical workup of each patient was taken by different ED physicians according to their clinical judgment and the availability of services, rather than shared protocols. The present review process was aimed at understanding how the different specialists approached each case, how the diagnosis was surmised, how the diagnosis was finally confirmed, and whether improvements in the system could be introduced. All patients were accompanied to the ED by a representative of the non-governmental organization (NGO) that had them in their charge.

All eight patients presented with hematuria. The ED physicians decided to refer five of them to the radiologist, one to the urologist, and three to the infectious disease specialist (Table 1). In one case, both the radiologist and infectious disease specialist were simultaneously summoned by the emergency physician. The first approach of both radiologists and urologists was the evaluation of the lower urinary tract (with ultrasonography [US] and cystoscopy, respectively).

**Table 1:** Demographic patient data and clinical presentation of patients with *Schistosoma haematobium* infection.

Patient	Nationality	Age, Years	Clinical Presentation	Specialist Who First Encountered the Patient
**1**	Mali	21	Hematuria	Radiologist
**2**	The Gambia	27	Hematuria	Infectious disease specialist
**3**	Guinea Bissau	25	Hematuria	Radiologist
**4**	Benin	20	Hematuria	Radiologist
**5**	Mali	30	Hematuria	Urologist
**6**	Mali	22	Hematuria	Radiologist
**7**	Italy	50	Microhematuria	Radiologist & infectious disease specialist
**8**	Ivory Coast	30	Hematuria	Infectious disease specialist

Seven patients had bladder abnormalities that were considered compatible with chronic SH infection [14] (Table 2). US showed focal thickening of the bladder wall and small parietal vegetations in four patients, with wall calcifications in one of these patients. Unenhanced computed tomography (CT) was performed in two cases: focal calcification on the posterior wall of the bladder was seen in one case, and diffuse calcification (involving the whole wall) was seen in the other. Thin, linear calcifications within the bladder wall are a “classical” finding, suggesting urinary SH infection. This patient was the first one encountered by the radiologist, who therefore called attention to the presence of this disease in the subsequent patients, who had less specific imaging findings [15].

**Table 2:** Laboratory, imaging, cystoscopic, and pathological findings of the eight patients presenting with *Schistosoma haematobium* infection.

Patient	Laboratory	Imaging	Cystoscopy	Histology
**1**	Anti-schistosoma Ab +	Two vegetations on the bladder wall, left hydroureteronephrosis on US, and diffuse linear calcification of the bladder wall on CT	Two broad-based mamillated lesions in the posterior bladder wall	Proliferative and granulomatous cystitis with erosive and polypoid aspects; partly calcified eggs and adult parasites
**2**	Anti-schistosoma Ab +	Negative	Negative	Not performed
**3**	Anti-schistosoma Ab +	Irregular bladder wall with three micronodular lesions and fine hyperechoic lines in the bladder wall on US	Lesions of the posterior bladder wall	Intense micropolypoid cystitis with rare calcified schistosome eggs
**4**	Not performed	Focal thickening and nodular lesions of the bladder wall on US; fine calcifications on CT	Hyperemic nodular areas with edema that bled easily involving the posterolateral bladder wall	Intense eosinophilic cystitis with partially calcified schistosome eggs
**5**	Anti-schistosoma Ab +	Not performed	Small hyperemic area in the posterior bladder wall with no evidence of active lesions; no biopsy	Not performed
**6**	Anti-schistosoma Ab +	Irregular bladder wall with one nodular lesion on US	Lesion of the posterior bladder wall	Intense eosinophilic acute cystitis, focally proliferative and microcystic, with numerous, rarely calcified, schistosome eggs
**7**	Anti-schistosoma Ab +	Negative	Not performed	Not performed
**8**	Anti-schistosoma Ab +	Negative	Negative; no biopsy	Not performed

Ab, antibody; US, ultrasonography; CT, computed tomography

**Fig 1:**
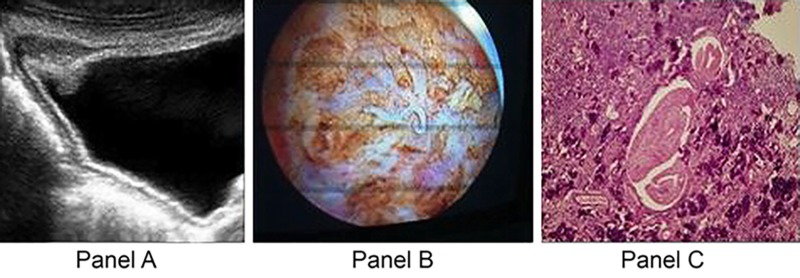
Representative ultrasonographic, cystoscopic, and pathological findings in a patient with chronic schistosomiasis presenting to the ED. (A) Ultrasonograph of the bladder shows a vegetation associated with bladder wall thickening; fine and diffuse calcifications are also visible. (B) Cystoscopic image obtained after transurethral resection shows an adult *S*. *haematobium* worm. (C) An area of marked, diffuse, acute inflammation with numerous eosinophils surrounds some calcified *S*. *haematobium* eggs; the parasite is also identified.

All eight patients with macroscopic hematuria underwent cystoscopic examination, as a first approach in one case and to exclude the presence of a tumor in the others. Cystoscopic findings were compatible with intermediate and late manifestations of SH infection (Fig 1).

Although SH infection may lead to squamous cell carcinoma of the bladder, this was not detected in this small series, which is in line with the reported low incidence of this complication (3–4/100,000) [16]. SH infection was confirmed pathologically in the five cases in which a biopsy specimen was obtained.

No bladder biopsy was performed in three cases that met the composite clinical and diagnostic criteria for SH infection (Table 2). In similar cases, as discussed above, availability of a more sensitive diagnostic technique for urinary SH antigen detection will provide useful information [12,13]. All patients were treated with praziquantel (40 mg/kg as a single dose).

Thus, our experience with the series described suggests that, in developed areas, young migrants from SH-endemic countries may predominantly have hematuria and may present to—or rather, may be accompanied by NGO representatives to—the ED; further, other differential diagnoses may primarily be considered, should anamnestic parameters (age and country of origin) not be adequately considered. Within this framework, there was an excess use of resources (US, CT, and emergency cystoscopy), both because SH infection was not considered in the first place and, more importantly, because a diagnostic workup commonly used for young European patients presenting with hematuria was employed.

## *Schistosoma haematobium*: Tropical—No more?

The number of international migrants worldwide has continued to grow rapidly over the past 15 years. Over the last year, this number has increased sharply, and the current migration patterns are likely to persist in the near future [17–18]. In view of the high migratory flux to European countries, this pattern of the clinical presentation of SH infection may more frequently be observed, resulting in an increased number of admissions to the ED and the need for greater awareness of this condition.

The recently described Corsica outbreak of SH infection in 2013 and the subsequent transmission occurring in 2015 after a bathing ban had been lifted [19–20] provided evidence that local transmission continues to occur. Autochthonous transmission is possible in parts of southern Europe where the intermediate host species, *Bulinus truncatus*, has been found [20–22]. This species has been identified in foci in Spain, Portugal, Sicily, and Greece. The fact that permissive species are reported in these areas (and that, in at least two of these areas, Sicily and Greece, a considerable number of migrants are concentrated upon arrival) could open the door to additional areas serving as regions where the transmission cycle is maintained locally. This possibility should raise concerns about the possible maintenance of the SH transmission cycle in Europe and provide a sense of urgency in addressing it. Interestingly, in line with this possibility, the SH isolates detected in the cases recorded in Corsica have been traced to a West African (Senegal) origin mixed with local animal schistosomes, based on molecular analyses [19]. These observations, therefore, suggest that SH infection in Europe should not be regarded as an issue affecting only immigrants or one that is devoid of potential concern for the native population in future. Indeed, the presence of intermediate snail hosts, together with the increasing flux from endemic areas, could potentially transfer SH endemicity to some areas in southern Europe. In this context, a scenario with increased inappropriate use of health services and underestimated and hidden costs, as described above, may represent an unacceptable additional burden of disease.

## Interventions and policy changes—Neglect no more?

Abandoning the present shortsighted or passive attitude in European countries regarding SH infection in migrant populations and the associated potential risks for both migrant and local populations may be difficult owing to the current limited awareness. The present case series highlights the fact that this disease is not considered a diagnostic possibility in European EDs, and this may lead to a misuse of health resources. Awareness could induce renewed efforts towards a more cost-effective approach.

Useful measures to constructively address this issue include educational, epidemiological, and clinical interventions. First, educational efforts have to target emergency and primary care physicians. Population changes need to be considered with increased awareness of SH infection symptoms and upgrading of diagnostic workup protocols to include not only symptoms but also disease prevalence in the country of origin. Increased involvement of infectious/tropical disease specialists, as well as widespread use of urine testing as a first diagnostic approach, will improve both the care of these populations and cost effectiveness. Second, a registry of emergency referrals to the ED of young adults from endemic countries would provide a real measure of the problem of SH infection in Europe. Distribution of urine tests to NGO facilities and testing of migrants on arrival would increase the accuracy of the estimation of the real prevalence of SH infection in this population. Further, a systematic survey of areas with the presence of suitable intermediate snail hosts could dismiss or confirm the risk for the transition to a local SH transmission cycle. In addition, geolocalization of SH infection cases in migrant populations may allow for assessment of the coexistence of SH-infected persons in areas where the intermediate snail host is known to be distributed, thus allowing for the estimation of the risk for local SH endemic transmission.

Finally, the introduction of MDA upon arrival in Europe in specific target populations may represent an innovative and cost-effective solution to decrease the long-term clinical complications of urogenital schistosomiasis and to reduce urinary egg elimination, thus helping to avoid a future persistence of SH infection in Europe. Identification of the optimal target population and reaching the identified target population should not be an issue among arriving migrants, thus allowing for campaign efficacy with high coverage rates [10–20].

In conclusion, SH infection is potentially set to become a stable presence in Europe, with a high likelihood of new prevalent cases associated with migration and the risk of a rising incidence, should establishment of endemicity occur in freshwater areas where intermediate snail hosts are already present. To improve the current efficiency of care, more attention should be paid to identification procedures and clinical protocols.

## References

[1] The Raven, Poe Edgar Allan. New York Evening Mirror; 29 1 1845.

[2] French MD, Churcher TS, Webster JP, Fleming FM, Fenwick A, Kabatereine NB, et al Estimation of changes in the force of infection for intestinal and urogenital schistosomiasis in countries with schistosomiasis control initiative-assisted programmes. Parasit Vectors. 2015;8: 558 10.1186/s13071-015-1138-1 26499981 PMC4619997

[3] Lai YS, Biedermann P, Ekpo UF, Garba A, Mathieu E, Midzi N, et al Spatial distribution of schistosomiasis and treatment needs in sub-Saharan Africa: a systematic review and geostatistical analysis. Lancet Infect Dis. 2015;15: 927–940. 10.1016/S1473-3099(15)00066-3 26004859

[4] Knowles SC, Webster BL, Garba A, Sacko M, Diaw OT, Fenwick A, et al Epidemiological interactions between urogenital and intestinal human schistosomiasis in the context of praziquantel treatment across three West African countries. PLoS Negl Trop Dis. 2015;9: e0004019 10.1371/journal.pntd.0004019 26469347 PMC4607489

[5] King CH, Sutherland LJ, Bertsch D. Systematic review and meta-analysis of the impact of chemical-based mollusciciding for control of Schistosoma mansoni and S. haematobium transmission. PLoS Negl Trop Dis. 2015;9: e0004290 10.1371/journal.pntd.0004290 26709922 PMC4692485

[6] World Health Organization. Schistosomiasis: number of people receiving preventive chemotherapy in 2012. Wkly Epidemiol Rec. No. 2, 2014;89: 21–28 24446558

[7] Chami GF, Kontoleon AA, Bulte E, Fenwick A, Kabatereine NB, Tukahebwa EM, et al Profiling nonrecipients of mass drug administration for schistosomiasis and hookworm infections: a comprehensive analysis of praziquantel and albendazole coverage in community-directed treatment in Uganda. Clin Infect Dis. 2016;62: 200–207. 10.1093/cid/civ829 26409064 PMC4690482

[8] World Health Organization. Schistosomiasis: progress report 2001–2011 and strategic plan 2012–2020. Geneva: World Health Organization; 2013 pp. 1–80.

[9] Poddighe D, Castelli L, Pulcrano G, Grosini A, Balzaretti M, Spadaro S, et al Urinary schistosomiasis in an adolescent refugee from Africa: an uncommon cause of hematuria and an emerging infectious disease in Europe. J Immigr Minor Health. 2016;18: 1237–1240. 10.1007/s10903-015-0272-3 26335551

[10] Cenderello G, Taramasso L, Riccardi N, Di Biagio A, Cassola G, De Maria A. Chemotherapy mass campaigns and migratory flows: an unexpected connection. Clin Infect Dis. 2016;62: 1323. 10.1093/cid/ciw087 26908811

[11] Monpierre O, Baudino P, Rio-René P, Maurice S, Malvy D, Receveur MC. [Global health of unaccompanied refugee minors in Gironde (France) between 2011 and 2013]. Bull Soc Pathol Exot. 2016;109: 99–106. 10.1007/s13149-016-0476-3 26860845

[12] Knopp S, Corstjens PL, Koukounari A, Cercamondi CI, Ame SM, Ali SM, et al Sensitivity and specificity of a urine circulating anodic antigen test for the diagnosis of Schistosoma haematobium in low endemic settings. PLoS Negl Trop Dis. 2015;9: e0003752 10.1371/journal.pntd.0003752 25973845 PMC4431728

[13] Ibironke O, Koukounari A, Asaolu S, Moustaki I, Shiff C. Validation of a new test for Schistosoma haematobium based on detection of Dra1 DNA fragments in urine: evaluation through latent class analysis. PLoS Negl Trop Dis. 2012;6: e1464 10.1371/journal.pntd.0001464 22235360 PMC3250497

[14] Shebel HM, Elsayes KM, Abou El Atta HM, Elguindy YM, El-Diasty TA. Genitourinary schistosomiasis: life cycle and radiologic-pathologic findings. Radiographics. 2012;32: 1031–1046. 10.1148/rg.324115162 22786992

[15] Skelly PJ. The use of imaging to detect schistosomes and diagnose schistosomiasis. Parasite Immunol. 2013;35: 295–301. 10.1111/pim.12040 23647173 PMC3766473

[16] Shiff C, Veltri R, Naples J, Quartey J, Otchere J, Anyan W, et al Ultrasound verification of bladder damage is associated with known biomarkers of bladder cancer in adults chronically infected with Schistosoma haematobium in Ghana. Trans R Soc Trop Med Hyg. 2006;100: 847–854. 10.1016/j.trstmh.2005.10.010 16443246

[17] Bijak J, Kupiszewska D, Kupiszewski M, Saczuk K, Kicinger A. Population and labour force projections for 27 European countries, 2002–052: impact of international migration on population ageing: Projections de population et de population active pour 27 pays européens 2002–052: impact de la migration internationale sur le vieillissement de la population. Eur J Popul. 2007. 10.1007/s10680-006-9110-6 PMC2798050 20076759

[18] United Nations Department of Economic and Social Affairs, Population Division. Trends in international migration, 2015. December 2015.

[19] Boissier J, Grech-Angelini S, Webster BL, Allienne JF, Huyse T, Mas-Coma S, et al Outbreak of urogenital schistosomiasis in Corsica (France): an epidemiological case study. Lancet Infect Dis. 2016;16: 971–979. 10.1016/S1473-3099(16)00175-4 27197551

[20] Berry A, Fillaux J, Martin-Blondel G, Boissier J, Iriart X, Marchou B, et al Evidence for a permanent presence of schistosomiasis in Corsica, France, 2015. Euro Surveill. 2016;21(1). 10.2807/1560-7917.ES.2016.21.1.30100 26767427

[21] Colley DG, Bustinduy AL, Secor WE, King CH. Human schistosomiasis. Lancet. 2014;383: 2253–2264. 10.1016/S0140-6736(13)61949-2 24698483 PMC4672382

[22] Brown DS. Freshwater snails of Africa and their medical importance. 1st ed London: Taylor and Francis; 1980.

